# A Novel 3D Reversible Data Hiding Scheme Based on Integer–Reversible Krawtchouk Transform for IoMT

**DOI:** 10.3390/s23187914

**Published:** 2023-09-15

**Authors:** Mohamed Yamni, Achraf Daoui, Paweł Pławiak, Haokun Mao, Osama Alfarraj, Ahmed A. Abd El-Latif

**Affiliations:** 1 Dhar El Mahrez Faculty of Science, Sidi Mohamed Ben Abdellah-Fez University, Fez 30000, Morocco; mohamed.yamni@usmba.ac.ma; 2 National School of Applied Sciences, Sidi Mohamed Ben Abdellah-Fez University, Fez 30000, Morocco; achraf.daoui@usmba.ac.ma; 3 Department of Computer Science, Faculty of Computer Science and Telecommunications, Cracow University of Technology, Warszawska 24, 31-155 Krakow, Poland; 4 Institute of Theoretical and Applied Informatics, Polish Academy of Sciences, Bałtycka 5, 44-100 Gliwice, Poland; 5 Information Countermeauser Technique Institute, Harbin Institute of Technology, School of Cyberspace Science, Faculty of Computing, Harbin 150001, China; hkmao@hit.edu.cn; 6 Computer Science Department, Community College, King Saud University, Riyadh 11437, Saudi Arabia; oalfarraj@ksu.edu.sa; 7 Department of Mathematics and Computer Science, Faculty of Science, Menoufia University, Shebeen El-Kom 32511, Egypt

**Keywords:** integer–reversible Krawtchouk transform (IRKT), reversible data hiding (RDH), internet of medical things (IoMT), secure storage and transmission

## Abstract

To avoid rounding errors associated with the limited representation of significant digits when applying the floating-point Krawtchouk transform in image processing, we present an integer and reversible version of the Krawtchouk transform (IRKT). This proposed IRKT generates integer-valued coefficients within the Krawtchouk domain, seamlessly aligning with the integer representation commonly utilized in lossless image applications. Building upon the IRKT, we introduce a novel 3D reversible data hiding (RDH) algorithm designed for the secure storage and transmission of extensive medical data within the IoMT (Internet of Medical Things) sector. Through the utilization of the IRKT-based 3D RDH method, a substantial amount of additional data can be embedded into 3D carrier medical images without augmenting their original size or compromising information integrity upon data extraction. Extensive experimental evaluations substantiate the effectiveness of the proposed algorithm, particularly regarding its high embedding capacity, imperceptibility, and resilience against statistical attacks. The integration of this proposed algorithm into the IoMT sector furnishes enhanced security measures for the safeguarded storage and transmission of massive medical data, thereby addressing the limitations of conventional 2D RDH algorithms for medical images.

## 1. Introduction

Integer-to-integer transforms (such as integer wavelet transforms [1], integer DCT [2], and integer color transform [3]) are an extension of traditional floating-point transforms. Unlike floating-point transforms that produce real-valued transformed coefficients, integer-to-integer transforms are specifically designed to directly generate integer-valued transformed coefficients. By exclusively operating on integer data throughout the entire transform process, including decomposition and reconstruction stages, these transforms eliminate round-off errors, computational overhead, and the need for quantization. Furthermore, they provide precise control over numerical accuracy. Integer-to-integer transforms have been studied and applied in lossless image compression [2,3,4,5,6] and image reversible data hiding (RDH) [7,8,9,10]. Such applications require accurate reconstruction of data without any loss of information. Various alternative variants of integer transforms have been investigated and developed, such as the integer discrete Fourier transform [11], integer fast Fourier transform [12], TS transform [5], the S + P transform [4], integer Tchebichef transform [6], and integer color transform [3].

In [13], the authors introduce for the first time another orthogonal transform called the Krawtchouk transform for digital image processing. This transform maps the input image data from the spatial domain to a different domain, called the Krawtchouk domain, defined by the Krawtchouk polynomials (kernel function of the transform). This transformed domain can provide valuable information about underlying patterns, structures, or features present in the data that may not be readily apparent in the original spatial representation. Furthermore, through the manipulation of control parameters within the framework of the Krawtchouk transform, it becomes possible to effectively capture and characterize local features present within an image. The Krawtchouk transform has demonstrated its utility in a range of image processing applications, including image reconstruction, image watermarking, and image zero-watermarking [13,14,15,16,17,18,19]. The authors in [13,14] have evaluated the Krawtchouk transform in image reconstruction and demonstrated that it outperforms several transforms (Tchebichef, Legendre, Zernike, and pseudo-Zernike transforms) in terms of reconstruction error. The authors in [15] proposed a geometrically invariant image watermarking scheme based on Krawtchouk transform. The authors in [17] proposed a Krawtchouk-transform-based watermarking scheme that automatically selects the optimum local region of the carrier image and embeds the watermark in the Krawtchouk domain. The authors in [19] proposed a robust blind zero-watermarking algorithm based on Krawtchouk radial transform, where the kernel function is the product of the Krawtchouk polynomials and an exponential function. The authors in [20] derived a new octonion transform called octonion Krawtchouk moment (OKM) transform that allows, in a compact and holistic way, global features to be simultaneously extracted from a set of seven images. And based on this transform, the authors introduced a new local zero-watermarking method for copyright protection of multiple medical images.

**Figure 1:**
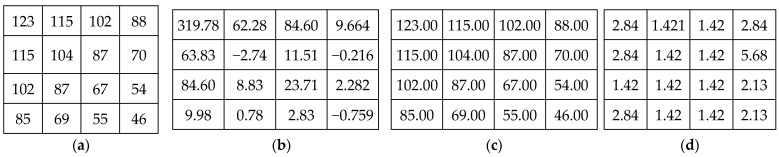
(**a**) Input block, (**b**) Krawtchouk transform, (**c**) reconstructed block, (**d**) absolute difference between the input block and the reconstructed block, factored by 10^−14^.

When applying the Krawtchouk transform on digital images that are represented as discrete pixel intensities (integer values), the output Krawtchouk coefficients are represented as floating-point numbers (i.e., real numbers) rather than integers (Figure 1). Consequently, the Krawtchouk transform is associated with some major problems, which are listed below:

- The original image cannot be perfectly reconstructed from the Krawtchouk coefficients, even if the Krawtchouk transform itself is orthogonal, due to rounding errors associated with the limited representation of significant digits. This can have an impact on the performance of lossless applications requiring exact calculations;
- Floating-point Krawtchouk coefficients require a more complex representation than integer image pixels (spatial domain), which means a higher cost in terms of memory and computing resources, especially when input images are large or voluminous (3D images). This can impact the performance of applications that require fast and efficient calculations;
- The Krawtchouk transform is unsuitable for applications where exact integer arithmetic is desired, such as lossless compression, RDH, digital communication systems, and embedded systems with limited precision.

**Figure 2:**
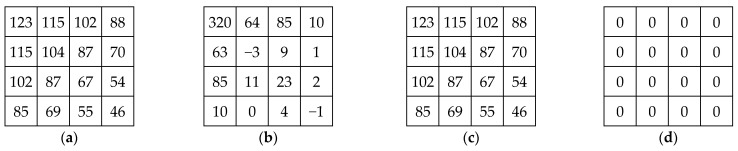
(**a**) Input block, (**b**) IRKT, (**c**) reconstructed block, (**d**) absolute difference input block and the reconstructed block.

Our primary goal in this paper is to propose a framework for deriving the integer–reversible Krawtchouk transform (IRKT) by using a SERM factorization [21] of the polynomial Krawtchouk matrix. The Krawtchouk polynomial matrix undergoes a decomposition process, during which it is decomposed into a series of Single-row Elementary Reversible Matrices (SERMs) and a permutation matrix. This decomposition is the key step in calculating the IRKT and its inverse (iIRKT). Instead of directly using the Krawtchouk polynomial matrix itself, SERMs and the permutation matrix are used to implement the Krawtchouk transform. Thanks to this decomposition, IRKT and iIRKT can be applied without generating computational errors or losing information (Figure 2).

As an illustration of the IRKT applications, an RDH algorithm for secure storage and transmission is investigated in the context of the Internet of Medical Things (IoMT). In the IoMT field (see the related works in Section 4), there is a massive amount of information that requires secure transmission and storage. The substantial volume of data renders conventional 2D RDH algorithms unsuitable due to their limited embedding capacity. This is attributed to the fact that 2D RDH solely employs a 2D image as a carrier, within which additional/secret data are concealed inside pixel intensities. To achieve heightened capacity, the most straightforward approach involves segmenting the additional/secret data and embedding them into several 2D medical images via the application of a 2D RDH algorithm, such as [22,23]. However, this strategy brings about disadvantages, including the iterative application of computational processes, resulting in notable time overhead, as well as the absence of compact storage and/or unified transmission of the additional/secret data as a single entity. An effective solution emerges in the development of a 3D RDH algorithm that utilizes 3D medical images as carriers, particularly when significant information needs to be sent or stored [24]. Through embracing the third dimension, 3D RDH techniques harness the potential of 3D image stacks, thereby facilitating the seamless embedding of concealed data across the volume. This eliminates the necessity for repetitive computations and introduces a streamlined mechanism for accommodating and transmitting the additional/secret data as a cohesive unit.

In recent years, with the development of 3D technology, more and more 3D medical images have been created and applied to each IoMT application, and also spread in a large amount by the network. Numerous 3D irreversible methods [25,26,27,28] and 3D RDH methods [29,30,31] have been proposed based on 3D image models, specifically using polygon mesh representation. However, these methods are not well suited for 3D medical images due to the limitations of polygon mesh representation, which often lack the ability to explicitly capture the intricate interior information present in such images.

Our second goal in this paper is to propose a novel 3D RDH algorithm for 3D grayscale medical images based on data integrity preservation and the reversibility of the proposed IRKT. The histogram-modification-based spatial approach described in [32] has been applied in the IRKT domain for the 3D RDH of medical images. The IKRT-based 3D RDH algorithm exhibits significant potential in terms of high embedding capacity, high additional data imperceptibility, and high robustness against statistical attacks.

As a summary, our main contributions are shown as follows:

- A precise, exact integer representation in the Krawtchouk domain is provided without rounding errors or accuracy limitations;
- IRKT guarantees that transformed coefficients remain integers (without the need for quantization), enabling lossless data reconstruction;
- The proposed IRKT can be easily generalized to accommodate 2D and 3D data representations;
- A novel 3D RDH algorithm suitable for 3D medical images is proposed, to the best of our knowledge, for the first time;
- Embedding data into 3D medical images does not increase their original size, thus optimizing infrastructure and maximizing resource utilization in the IoMT;
- Medical images are recovered without any loss or damage after extracting additional data;
- The embedding capacity and quality of the 3D stego image (image after data embedding) can be adjusted using a threshold-based embedding technique.

The rest of the paper is organized as follows. Some preliminaries on the Krawtchouk transform are provided and its limit is discussed in Section 2. In Section 3, the detailed procedure for deriving IRKT and some properties are provided. The 3D RDH algorithm for 3D grayscale medical images is presented in Section 4. Experiments designed to demonstrate its effectiveness are carried out in Section 5, and Section 6 concludes the paper.

## 2. Preliminaries

The Krawtchouk transform of a 1D signal *f*(*x*) of length *N* is defined as [13]

(1) $$Q_{n}=\sum_{x=0}^{N-1}K_{n}(x;p,N-1)f(x),\;n=0,1,\ldots ,N-1$$

where *Q_n_* is the Krawtchouk transform of *f*(*x*), and *K_n_*(*x*; *p*, *N*) is the Krawtchouk polynomials of degree *n*:

(2) $$K_{n}(x;p,N-1)={}_{2}F_{1}(-n,-x;-N+1;\frac{1}{p})\;\sqrt{{\left(-1\right)}^{n}{\left(-N+1\right)}_{n}\left(\begin{array}{c} N-1\\ x \end{array}\right)\frac{p^{n+x}{\left(1-p\right)}^{N-x-n-1}}{n!}}$$

where *x*, *n* = 0, 1, …, *N* − 1, p∈(0,1) is the Krawtchouk parameter controlling the shape of the polynomial, _2_*F*_1_ represents the hypergeometric function, which can be defined as

(3) $${}_{2}F_{1}(a,b;c;z)=\sum_{k=0}^{\infty }\frac{{\left(a\right)}_{k}{\left(b\right)}_{k}}{{\left(c\right)}_{k}}\frac{z^{k}}{k!}$$

and (*a*)*_k_* is the Pochhammer symbol, defined as

(4) $$\begin{array}{l} {\left(a\right)}_{0}=1\\ {\left(a\right)}_{k}=a(a+1)(a+2)\ldots (a+k-1),\;k\geq 1 \end{array}$$

The Krawtchouk polynomials exhibit a distinct property of orthogonality, as stated in [13]

(5) $$\sum_{x=0}^{N-1}K_{n}(x;p,N-1)K_{m}(x;p,N-1)=\delta_{n,m}$$

The property of orthogonality gives rise to the subsequent inverse Krawtchouk transform as follows [13]:

(6) $$f(x)=\sum_{n=0}^{N-1}Q_{n}K_{n}(x;p,N-1)$$

In the case of an image *f*(*x*, *y*) of size *N* × *N*, the two-dimensional Krawtchouk transform of order *n* + *m* and its inverse can be expressed mathematically as follows:

(7) $$Q_{n,m}=\sum_{x=0}^{N-1}\sum_{y=0}^{N-1}K_{n}(x;p,N-1)K_{m}(y;p,N-1)f(x,y)$$

(8) $$f(x,y)=\sum_{n=0}^{N-1}\sum_{m=0}^{N-1}Q_{n,m}K_{n}(x;p,N-1)K_{m}(y;p,N-1)$$

In the scenario of an *N* × *N* × *N* volumetric image *f*(*x*, *y*, *z*), the three-dimensional Krawtchouk transform and its inverse can be mathematically represented as follows [33]:

(9) $$Q_{n,m,\ell }=\sum_{x=0}^{N-1}\sum_{y=0}^{N-1}\sum_{z=0}^{N-1}K_{n}(x;p,N-1)K_{m}(y;p,M-1)K_{\ell }(z;p,N-1)f(x,y,z)$$

(10) $$f(x,y,z)=\sum_{n=0}^{N-1}\sum_{m=0}^{N-1}\sum_{\ell =0}^{N-1}Q_{n,m,\ell }K_{n}(x;p,N-1)K_{m}(y;p,N-1)K_{\ell }(z;p,N-1)$$

When original integer data are transformed into the Krawtchouk domain, they are represented by a set of floating-point coefficients (real numbers) resulting from the projection of data onto the Krawtchouk polynomials. However, due to factors such as numerical precision or approximation errors during the Krawtchouk transform, the original data cannot be perfectly reconstructed from these coefficients. For example, Figure 1 shows an input data block, its Krawtchouk transform, the reconstructed block via the inverse Krawtchouk transform, and the absolute difference between the original and reconstructed blocks. The calculation is based on the IEEE Standard 754 for double precision. It is clear that the reconstructed block is not exactly the original input block, which means that information is lost in this process, even though the Krawtchouk transform itself is orthogonal (Equation (5)). In lossless image applications like reversible data hiding (RDH), lossless compression, or lossless watermarking, preserving the exact values and maintaining data integrity is crucial. Therefore, the Krawtchouk transform is not suitable for these applications where any information loss is not acceptable. In order to overcome this limitation, we propose in the next section an integer and reversible version of the Krawtchouk transform that is more suitable for lossless image applications.

## 3. Construction of the Integer–Reversible Krawtchouk Transform

In this section, we propose an integer–reversible Krawtchouk transform (IRKT), which can be applied on integer data and generate integer Krawtchouk coefficients (integer-based representation), and its inverse IRKT can exactly restore the original data from the Krawtchouk coefficients. The Krawtchouk polynomial matrix undergoes a decomposition process, during which it is decomposed into a series of Single-row Elementary Reversible Matrices (SERMs) and a permutation matrix. This decomposition is the key step in calculating the IRKT and its inverse (iIRKT). Instead of directly using the Krawtchouk polynomial matrix itself, SERMs and the permutation matrix are used to implement the Krawtchouk transform. Thanks to this decomposition, IRKT and iIRKT can be applied without generating computational errors or loss of information.

### 3.1. SERM Factorization of the Krawtchouk Polynomial Matrix

Based on [21], it has been established that for any nonsingular *N* × *N* matrix ***A***, a unit SREM factorization of ***A*** = ***S****_N_*…***S***_2_***S***_1_ exists if and only if all the minors of the leading principal submatrices of ***A*** are ones, where ***S****_j_*(*j* = 0, 1, 2,…, *N*) represents the SERMs involved in the factorization. In particular, if the determinant of ***A*** is an integer number, then an *N* + 1 SERM factorization ***A*** = ***PS****_N_*…***S***_2_***S***_1_***S***_0_ can be obtained. This factorization involves the use of permutation matrix ***P*** and unit SERMs ***S****_j_*(*j* = 0, 1, 2, …, *N*).

The SERM factorization of the Krawtchouk polynomial matrix can be obtained as follows.

The matrix representation of the 1D Krawtchouk transform, which applies to a signal *f*(*x*) of length *N* as defined in Equation (1), can be expressed as

(11) Q=Kf

where ***K*** is the Krawtchouk polynomial matrix defined as follows:

(12) $$K_{n,x}=K_{n}(x;p,N-1),n,x=0,1,\ldots ,N-1$$

Typical Krawtchouk polynomial matrices of size 4 × 4 and 8 × 8 for Krawtchouk transform and its inverse are as follows:

$$K_{4\times 4}=\left(\begin{array}{rrrr} 0.3536 & 0.6124 & 0.6124 & 0.3536\\ 0.6124 & 0.3536 & -0.3536 & -0.6124\\ 0.6124 & -0.3536 & -0.3536 & 0.6124\\ 0.3536 & -0.6124 & 0.6124 & -0.3536 \end{array}\right)\;K_{8\times 8}=\left(\begin{array}{rrrrrrrr} 0.0884 & 0.2339 & 0.4050 & 0.5229 & 0.5229 & 0.4050 & 0.2339 & 0.0884\\ 0.2339 & 0.4419 & 0.4593 & 0.1976 & -0.1976 & -0.4593 & -0.4419 & -0.2339\\ 0.4050 & 0.4593 & 0.0884 & -0.3423 & -0.3423 & 0.0884 & 0.4593 & 0.4050\\ 0.5229 & 0.1976 & -0.3423 & -0.2652 & 0.2652 & 0.3423 & -0.1976 & -0.5229\\ 0.5229 & -0.1976 & -0.3423 & 0.2652 & 0.2652 & -0.3423 & -0.1976 & 0.5229\\ 0.4050 & -0.4593 & 0.0884 & 0.3423 & -0.3423 & -0.0884 & 0.4593 & -0.4050\\ 0.2339 & -0.4419 & 0.4593 & -0.1976 & -0.1976 & 0.4593 & -0.4419 & 0.2339\\ 0.0884 & -0.2339 & 0.4050 & -0.5229 & 0.5229 & -0.4050 & 0.2339 & -0.0884 \end{array}\right)$$

The matrix ***K*** satisfies the following properties:

- Property 1: ***K*** is orthogonal, that is, ***KK****^T^* = ***K****^T^**K** = **I***, ***K****^T^* is the transpose of ***K***, and ***I*** is the identity matrix [13] of the appropriate size;
- Property 2: ***K*** is nonsingular, that is, ***KK***^−1^ = ***K***^−1^***K*** = ***I***;
- Property 3: The determinant of ***K*** is det(***K***) = 1;
- Property 4: The minors of the leading principal submatrices of ***K*** are all ones.

According to the Corollary 6 in [21], the previous properties of ***K*** lead to the following SERM factorization:

(13) $$K=PS_{N}\ldots S_{2}S_{1}S_{0}$$

where ***P*** is the permutation matrix of ***K***, and ***S****_j_*(*j* = 0, 1,…, *N*) are SERMs. An SERM represents a square matrix of dimension *N × N*, wherein the diagonal is composed of integer factors, and solely one row possesses off-diagonal elements that are not entirely composed of zeros. The SERM matrix ***S****_j_* can be expressed as

(14) $$\begin{array}{l} S_{0}=I+e_{N}s_{0}^{T}\\ S_{j}=I+e_{j}s_{j}^{T}\;,\;j=1,2,\ldots ,N \end{array}$$

The inverse of ***S****_j_* can be expressed as follows:

(15) $$\begin{array}{l} S_{0}^{-1}=S_{0}=I+e_{N}s_{0}^{T}\\ S_{j}^{-1}=I-e_{j}s_{j}^{T},\;j=1,2,\ldots ,N \end{array}$$

where ***I*** is the identity matrix of size *N × N*, ***e****_j_* refers to the column vector corresponding to the *j*-th column of ***I***, and ***s****_j_* is a vector whose *j*-th element is zero.

The permutation matrices of ***K*** for sizes 4 × 4 and 8 × 8 are as follows:

$$P_{4\times 4}=\left(\begin{array}{l} \;0\;1\;0\;0\\ \;1\;0\;0\;0\\ \;0\;0\;0\;1\\ \;0\;0\;1\;0 \end{array}\right)\;P_{8\times 8}=\left(\begin{array}{l} \;0\;0\;0\;1\;0\;0\;0\;0\\ \;0\;0\;1\;0\;0\;0\;0\;0\\ \;0\;0\;0\;0\;0\;0\;1\;0\\ \;0\;0\;0\;0\;1\;0\;0\;0\\ \;1\;0\;0\;0\;0\;0\;0\;0\\ \;0\;0\;0\;0\;0\;1\;0\;0\\ \;0\;1\;0\;0\;0\;0\;0\;0\\ \;0\;0\;0\;0\;0\;0\;0\;1 \end{array}\right)$$

The row vectors for computing the SERMs of ***K*** for sizes 4 × 4 and 8 × 8 are listed in Table 1 and Table 2, respectively.

### 3.2. Integer–Reversible Krawtchouk Transform

By utilizing the SERM factorization of the Krawtchouk polynomial matrix ***K***, as discussed in the previous subsection, we can establish an integer–reversible version of the Krawtchouk transform (IRKT).

For an integer vector ***f*** = (*f_0_*, *f_1_*,…, *f_N_*)*^T^* of length *N*, the 1D IRKT can be defined as

(16) $$Q=IRKT(f)=P\left\lfloor S_{N}\ldots \left\lfloor S_{1}\left\lfloor S_{0}f\right\rfloor \right\rfloor \ldots \right\rfloor$$

where ⌊.⌋ is the rounding arithmetic, ***S****_j_*(*j* = 0, 1,…, *N*) are SERMs of the *N × N* matrix ***K***, and ***P*** is the permutation matrix of ***K***.

The corresponding inverse IRKT (iIRKT) can be expressed as

(17) $$f=iIRKT(Q)=\left\lfloor S_{0}^{-1}\left\lfloor S_{1}^{-1}\ldots \left\lfloor S_{N}^{-1}P^{T}Q\right\rfloor \ldots \right\rfloor \right\rfloor$$

To compute the 2D IRKT for an *N* × *N* block $f={\left\{f(x,y)\right\}}_{x,y=0}^{x,y=N-1}$, the 1D IRKT is firstly applied to each column of the block. This operation transforms each column individually. After that, the 1D IRKT is then applied to each row of the resulting transform obtained from the previous step. This process ensures that the transform is performed on both the columns and rows, completing the 2D IRKT for the *N × N* block. The mathematical formula of 2D-IRKT can be represented as

(18) $$Q=IRKT\left({\left(IRKT(f)\right)}^{T}\right)$$

and the 2-D inverse IRKT as

(19) $$f=iIRKT\left({\left(iIRKT(Q)\right)}^{T}\right)$$

Figure 2 shows a block of input data (the same block used in Figure 1), its IRKT, the block reconstructed via iIRKT, and the absolute difference between the original and reconstructed blocks. It is clear that the reconstructed block is exactly the original input block, meaning that the IRKT can provide an exact reconstruction of the original data.

The IRKT can be easily generalized to 3D situations as follows. To compute the 3D IRKT for an *N × N × N* volumetric cuboid, a stepwise approach is employed as follows: (1) First, the 1D IRKT is applied to each column of every 2D plane in the cuboid. This operation transforms each column within each 2D plane individually. (2) Next, the 1D IRKT is applied to each row of every 2D plane in the transformed cuboid obtained from the previous step. This operation transforms each row within each 2D plane. (3) Finally, the 1D IRKT is applied to each depth plane of the cuboid. This operation transforms each individual depth plane.

Detailed descriptions for implementing 3D IRKT and inverse 3D IRKT are given in Algorithm 1 and Algorithm 2, respectively.
**Algorithm 1** 3D Integer–Reversible Krawtchouk Transform.**Input: *C*** a volumetric cuboid of size *N × N × N*, ***P*** the permutation matrix of ***K***, ***S****_j_*(*j* = 0, 1,…, *N*) the SERMs of ***K***.
**Output:** Transformed cuboid ***Q*** of size *N × N × N*
Initialize empty matrices ***Q***, ***Q_1_***, ***Q_2_*** of size *N × N × N*.
**for** *i* = 1 to *N* **do**

   ***A*** = ***P***⌊***S****_N_*⌊…⌊ ***S***_1_⌊***S***_0_***C***(:,:,*i*) ⌋⌋…⌋;//***C***(:,:,*i*) is the *i*-th plane along the *z*-axis.
   ***Q_1_***(:,:,*i*) = **P**⌊***S****_N_*⌊…⌊ ***S***_1_⌊***S***_0_***A****^T^*⌋⌋…⌋;//***A****^T^* is the transpose of ***A***.
**end**
//Transpose the 3D matrix ***Q_1_*** into another 3D matrix ***Q_2_*.**
**for** *i* = 1 to *N* **do**
   **for** *j* = 1 to *N* **do**
     ***Q_2_***(*j*,:,*i*) = ***Q_1_***(*i*,:,*j*); 
   **end**

**end**
//Generate the IRKT matrix ***Q***
**for** *i* = 1 to *N* **do**
   ***A*** = ***P***⌊***S****_N_*⌊…⌊***S***_1_⌊***S***_0_***Q_2_***(:,:,*i*) ⌋⌋…⌋;
   ***Q***(:,:,*i*) = ***P***⌊***S****_N_*⌊…⌊***S***_1_⌊***S***_0_***A****^T^*⌋…⌋;
**end**
**Return** the IRKT matrix ***Q***.

**Algorithm 2** Inverse 3D Integer–Reversible Krawtchouk Transform.**Input:** IRKT matrix***Q*** of size *N × N × N*,***P*** the permutation matrix of ***K***, ***S****_j_*(*j* = 0, 1,…, *N*) the SERMs of ***K***.
**Output:** Reconstructed cuboid ***R*** of size *N × N×N*.
Initialize empty matrices ***R***, ***R_1_***, ***R_2_*** of size *N × N × N*.
Compute $S_{j}^{-1}$ the inverse of ***S****_j_*.
**for** *i* = 1 to *N* **do**
   ***A***= ⌊$S_{0}^{-1}$⌊$S_{1}^{-1}$…⌊$S_{N}^{-1}$***P****^T^****Q***(:,:,*i*)⌋…⌋⌋;//***P****^T^* is the transpose of ***P*.**
   ***R_1_***(:,:,*i*) = ⌊$S_{0}^{-1}$⌊$S_{1}^{-1}$…⌊$S_{N}^{-1}$***P****^T^****A****^T^*⌋…⌋⌋; 
**end**
**for** *i* = 1 to *N* **do**
   **for** *j* = 1 to *N* **do**
     ***R_2_***(*j*,:,*i*) = ***R_1_***(*i*,:,*j*);
   **end**
**end**
**for** *i* = 1 to *N* **do**
   ***A***= ⌊$S_{0}^{-1}$⌊$S_{1}^{-1}$…⌊$S_{N}^{-1}$***P****^T^****R_2_*** (:,:,*i*)⌋…⌋⌋;
   ***R***(:,:,*i*) = $S_{0}^{-1}$⌊$S_{1}^{-1}$…⌊$S_{N}^{-1}$***P****^T^****A****^T^*⌋…⌋⌋;
**end**
**Return** the Reconstructed cuboid ***R***.

## 4. Application in RDH for 3D Medical Images

The proposed IRKT can be applied in many lossless applications such as lossless compression, lossless encryption, and lossless watermarking. Due to the limited paper length, the reversible data hiding (RDH) application is only discussed in the context of the Internet of Medical Things (IoMT).

Schematically, RDH is a practical lossless technique that can be mainly used to ensure secure transmission and storage of data [32]. This can be accomplished by embedding additional data (secret or sensitive data), in an imperceptible manner, into an image (carrier image) while ensuring the complete recovery of the original image without any loss after data extraction [34,35,36,37,38,39,40]. RDH techniques can be classified into three categories: space-based techniques, compression-based techniques, and transform-domain-based techniques [9]. Since space- and compression-based techniques embed the additional data into the carrier image directly, they are relatively weak under statistical attacks [9]. In contrast, transform-domain-based methods generally provide an additional layer of security and are relatively more resistant to statistical attacks [41].

Recently, a number of data hiding methods based on moment transforms [42,43,44,45,46] have been proposed. However, it is important to note that these methods are irreversible since they embed additional data into the real-valued moment transform domain. Consequently, these methods result in a loss of information from the original image, making it nonrecoverable. This is why the integer–reversible transforms have been more widely studied in RDH applications. Noteworthy examples of such transforms include the integer wavelet transforms [8,9] and integer DCT [10].

Among the RDH strategies that are simple to implement and require a short execution time, histogram modification is proposed by Ni et al. [32]. The authors embedded the additional data by modifying the histogram of the carrier image. In addition to its simplicity, this strategy produces a stego image of high quality. Ni’s strategy has been successfully adopted by several methods in the transform domain [9,10,47,48,49,50].

In the IoMT field (See Section 4.1), the amount of information requiring secure transmission and storage is gigantic/huge, making the conventional 2D RDH algorithm for medical images unsuitable due to its limited embedding capacity. Indeed, the data that can be embedded into the carrier medical image are the Electronic Patient Record (EPR), diagnostic annotations, measurements, or any other additional details that can contribute to more accurate and informed diagnoses and decisions.

To achieve high capacity, the most straightforward practice is to divide the additional data, and then embed them in several 2D medical images by applying the 2D RDH algorithm, such as [22,23]. However, this approach has the disadvantages of applying the computation scheme several times, adding significant time overhead, and the additional data are not stored and/or sent compactly in a single entity. An effective solution is to develop a 3D RDH algorithm that uses 3D medical images as carriers when a large amount of information needs to be sent or stored [24].

In recent years, with the development of 3D technology, more and more 3D medical images are created and applied to each IoMT application, and are also spread in a large amount by the network.

Numerous 3D irreversible methods [25,26,27,28] and 3D RDH methods [29,30,31] have been proposed based on 3D image models, specifically using polygon mesh representation. However, these methods are not well suited for 3D medical images. While polygonal models can effectively represent the surfaces of objects and scenes in 3D space, they often lack explicit capture of interior information. In contrast, voxel-based representation is commonly employed in 3D medical imaging, where each voxel represents a small volume element and stores information about the object at that specific location. This representation allows for the inclusion of both exterior and interior details, making it more suitable for accurately representing the complex structures and characteristics found in 3D medical images.

In this section, we propose a new 3D RDH scheme for 3D medical images based on data integrity preservation and reversibility of the proposed IRKT.

### 4.1. The Usefulness of 3D RDH in IoMT

The IoMT is a burgeoning field that encompasses the integration of medical devices, sensors, and healthcare systems with the internet. It enables the seamless exchange, analysis, and management of healthcare data, revolutionizing the delivery of medical services [51]. The IoMT has the potential to enhance patient care, facilitate remote monitoring, enable real-time interventions, and improve healthcare outcomes. However, the extensive connectivity and data sharing in IoMT also raise concerns regarding privacy, data security, data transmission and storage, and efficient data utilization. The RDH technique allows additional information to be hidden within medical images without compromising their visual quality, ensuring secure storage and transmission while preserving patient privacy.

The utilization of 3D RDH techniques, employing 3D medical images as carrier images, provides distinct advantages in IoMT applications:

- Due to the substantial volume of data generated by IoMT, storage and transmission may encounter limitations. By storing and transmitting a significant amount of medical data within the same carrier image, efficient resource utilization and management can be achieved within IoMT applications;
- Protecting patient privacy is crucial. The 3D RDH can selectively embed confidential patient identifiers or sensitive information within 3D medical images, ensuring that only authorized personnel can access these details;
- By embedding additional diagnostic information into 3D medical images, the accuracy of diagnoses can be enhanced, thereby granting healthcare professionals extended access to relevant data during remote consultations;
- In telemedicine scenarios, real-time interaction is limited. The 3D RDH can hide additional explanations, annotations, or visual cues within 3D medical images, offering remote healthcare professionals a more detailed understanding of the patient’s condition;
- In the context of IoMT, medical data are frequently employed for research and analysis purposes. The adoption of 3D RDH alongside 3D medical images enables the integration of research data, metadata, or annotations into the images. This facilitates comprehensive data analysis, data exploration, and collaboration among researchers, thereby enabling them to gain deeper insights into medical conditions and treatment outcomes.

In the following, the histogram-modification-based spatial approach described in [32] has been applied in the IRKT domain for 3D RDH of medical images. The IRKT-based 3D RDH algorithm has the following features: (1) it is an integer-value-to-integer-value mapping in both data embedding and data extraction processes; (2) it exhibits significant potential in terms of high embedding capacity, high additional data imperceptibility, and high robustness against statistical attacks; (3) embedding data into 3D medical images does not increase their original size, thus optimizing infrastructure and maximizing resource utilization in the IoMT; (4) medical images are recovered without any loss or damage after extracting additional information; (5) it is well suited for high-quality 3D medical images; (6) the embedding capacity and quality of the 3D stego image (image after data embedding) can be adjusted using a threshold-based embedding technique; (7) partitioning the 3D carrier image into smaller cuboids offers practical advantages in terms of implementation and algorithm complexity reduction.

The embedding, extraction, and restoration procedures are described in the following subsections.

### 4.2. Embedding Procedure

**Figure 3:**
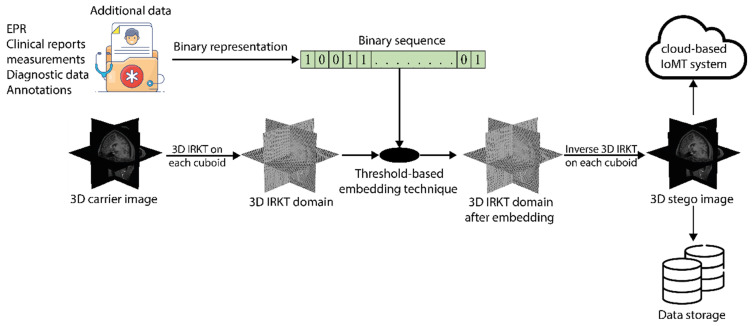
General flowchart of the proposed 3D RDH in IRKT domain.

Consider a 3D medical image $f={\left\{f(x,y,z)\right\}}_{x,y,z=0}^{x,y,z=N-1}$ with *N* × *N* × *N* voxels. The additional data are embedded into the carrier image ***f***, as depicted in Figure 3, through a series of steps, which are outlined below:

Step 1: Encode the additional data into a binary representation. We refer to the resulting binary vector of length *L* as $V=\left\{V_{\ell },\;\ell =0,1,\ldots ,L-1\right\}$. Here, the additional data are the Electronic Patient Record (EPR), diagnostic annotations, measurements, or any other additional details that can contribute to more accurate and informed diagnoses and decisions.

Step 2: The 3D carrier image ***f*** is divided into *n* × *n* × *n* nonoverlapping volumetric cuboids, and the 3D IRKT (Algorithm 1) is computed for each cuboid. Then, the IRKT cuboids are collected into a single 3D matrix of size *N* × *N* × *N*, denoted by ***Q***.

Step 3: Create an empty space in the matrix ***Q*** to insert the additional data. Here, the term “empty space” refers to the values of the IRKT coefficients being modified by replacing their original values. The size of the empty space determines the amount of data that can be inserted. To control and adjust the amount of data to be inserted, we employ a threshold-based embedding technique. A positive integer value, T>0, is used to adjust the embedding capacity and the quality of the stego image.

Let us consider ***Q***(*i*, *j*, *k*) as the value of the IRKT coefficient at coordinate (*i*, *j*, *k*), and its modified value denoted by ***Q′***(*i*, *j*, *k*). The modification follows the rules

(20) $$Q^{\prime }(i,j,k)=\left\{ \begin{array}{ll} Q(i,j,k)+T & \;\mathrm{if}\;Q(i,j,k)\geq T\\ Q(i,j,k)-T & \;\mathrm{if}\;Q(i,j,k)<T\\ Q(i,j,k) & \;\mathrm{otherwise} \end{array}\right.$$

where $Q^{\prime }=\{Q^{\prime }(i,j,k),\;0\leq i,j,k<N\}$ represents the modified IRKT matrix. From Equation (20), all the IRKT coefficients with values satisfying Q(i,j,k)≥T are increased by *T*, while those with values satisfying Q(i,j,k)<−T are decreased by *T*. The values in the interval [−*T*, *T*) are not modified.

By applying this step, an empty space, in [−2*T*, −*T*) and [*T*, 2*T*), is created within the matrix ***Q′***, allowing for the subsequent embedding of the additional data.

Step 4: In the proposed method, the number of additional data bits to be embedded is equal to the number of IRKT coefficients in the range [−*T*, *T*). The matrix ***Q′*** is scanned, and when IRKT coefficients, whose value ***Q′***(*i*, *j*, *k*) is in the interval [−*T*, *T*), are encountered, they are modified according to the embedded bit values. The rule for embedding $\left\{V_{\ell }\right\}$ is as follows:

(21) $$Q_{s}(i,j,k)=\left\{ \begin{array}{ll} Q^{\prime }(i,j,k)+T & \;\mathrm{if}\;0\leq Q^{\prime }(i,j,k)<T\;\&\;V_{\ell }=1\\ Q^{\prime }(i,j,k)-T & \;\mathrm{if}\;-T\leq Q^{\prime }(i,j,k)<0\;\&\;V_{\ell }=1\\ Q^{\prime }(i,j,k) & \;\mathrm{if}\;-T\leq Q^{\prime }(i,j,k)<T\;\&\;V_{\ell }=0\\ Q^{\prime }(i,j,k) & \;\mathrm{if}\;Q^{\prime }(i,j,k)<-Tor\;Q^{\prime }(i,j,k)\geq T \end{array}\right.$$

where $Q_{s}=\{Q_{s}(i,j,k),\;0\leq i,j,k<N\}$ represents the stego IRKT matrix.

Through this step, the empty space in the intervals [−2*T*, −*T*) and [*T*, 2*T*) is effectively filled within the matrix ***Q****_s_*. Negative IRKT coefficients in the interval [−*T*, *T*) are moved into the empty interval [−2*T*, −*T*) and positive ones into the empty interval [*T*, 2*T*) if $V_{\ell }=1$. If not, the IRKT coefficients remain unchanged.

Step 5: The 3D inverse IRKT (Algorithm 2) is applied to each stego cuboid of size *n* × *n* × *n* within the matrix ***Q****_s_* to obtain the stego image ***f****_s_*.

The output stego image, which contains the additional data, can be effectively transmitted or securely stored within the IoMT ecosystem. In both cases, the additional data remain in the stego image. Authorized parties can extract these data and recover the original image from the stego image by running the decoding algorithm presented in the next section.

### 4.3. Additional Data Extraction and Carrier Image Restoration

Step 1: The 3D stego image ***f****_s_* is divided into nonoverlapping cuboids of size *n* × *n* × *n*. The resulting IRKT cuboids are combined into a matrix denoted by ***Q****_s_*.

Step 2: During the extraction of additional data, ***Q****_s_* is scanned as in the embedding procedure, and then the sequence $\left\{V_{\ell }\right\}$ is extracted from ***Q****_s_* using the following rule:

(22) $$V_{\ell }=\left\{ \begin{array}{l} 0\;\mathrm{if}\;-T\leq Q_{s}(i,j,k)<T\;\\ 1\;\mathrm{if}\;-2T\leq Q_{s}(i,j,k)<-T\;or\;T\leq Q_{s}(i,j,k)<2T \end{array}\right.$$

Step 3: During the restoration of the carrier image, the IRKT coefficients that were modified during embedding steps 3 and 4 are reverted to their original values. This is accomplished as follows:

(23) $$Q(i,j,k)=\left\{ \begin{array}{l} Q_{s}(i,j,k)+T\;\mathrm{if}\;Q_{s}(i,j,k)<-T\\ Q_{s}(i,j,k)-T\;\mathrm{if}\;Q_{s}(i,j,k)\geq 0\\ Q_{s}(i,j,k)\;\mathrm{otherwise} \end{array}\right.$$

where Q={Q(i,j,k), 0≤i,j,k<N} represents the original carrier IRKT matrix.

Step 4: Finally, the inverse IRKT is applied on ***Q*** to restore the original carrier image.

The efficiency of the IRKT-based 3D RDH algorithm in terms of embedding capacity and 3D stego image quality can be improved by adjusting the threshold *T* in the IRKT domain, the Krawtchouk parameter p∈(0,1), and the cuboid size used for 3D IRKT computation. The following section discusses the influence of these parameters on the algorithm.

## 5. Experimental Results

A series of experiments were conducted to assess the efficiency of the 3D RDH algorithm based on IRKT for medical images. The algorithm was implemented using Matlab R2022b on a computing system equipped with an AMD Ryzen 5 5600U CPU, which has six cores running at 2.30 GHz and 8 GB of RAM. In the conducted experiments, a set of 208 grayscale 3D medical images obtained from the LIDC-IDRI [52], OpenNeuro [53], IRCAD [54], and BraTS 2021 [55] datasets served as the carrier images. Simultaneously, a randomly generated sequence of binary digits {0, 1} was employed as the additional data intended for embedding. More details on the test datasets are provided in Table 3. This dataset comprises 3D medical images encompassing both CT and MRI modalities, including both signed and unsigned images, with a depth of 16 bits (indicating high quality). The images are stored in DICOM and NIfTI formats. On average, it took 4.5 s, 8.7 s, 13.65 s, and 21.41 s to embed additional data bits into 3D medical images of sizes 96 × 96 × 96, 128 × 128 × 128, 160 × 160 × 160, and 192 × 192 × 192, respectively.

This IRKT-based RDH algorithm is compared with the spatial approach reported in [29,30,31,32,56], as well as the RDH using integer DCT [10], integer wavelet transforms [8,9], standard DCT [57], and standard DWT [58].

The algorithm’s performance in terms of additional data imperceptibility is quantified by the peak signal-to-noise ratio (PSNR) defined as follows.

(24) $$\mathrm{PSNR}(\mathrm{dB})=10\log_{10}\left(\frac{Max^{2}}{\mathrm{MSE}}\right)$$

where *Max* is the maximum possible value for a voxel in the image, and MSE is the mean squared error between the carrier and stego images:

(25) $$\mathrm{MSE}=\frac{1}{M\times N\times K}\sum_{x=1}^{M-1}\sum_{y=1}^{N-1}\sum_{z=1}^{K-1}{\left[f(x,y,z)-f_{s}(x,y,z)\right]}^{2}$$

where *f* and *f_s_* are the carrier image and stego one of size *M* × *N* × *K*, respectively.

The amount of information carried by the 3D stego image, known as embedding capacity (EC), is measured in bits per voxel (bpv), and defined as follows:

(26) $$\mathrm{EC}(\mathrm{bpv})=\frac{\mathrm{Additional}\;\mathrm{data}\;\mathrm{size}\;(\mathrm{bits})}{\mathrm{Total}\;\mathrm{number}\;\mathrm{of}\;\mathrm{voxels}\;\mathrm{in}\;3D\;\mathrm{carrier}\;\mathrm{image}}$$

In the following experiments, the cuboid size used for 3D IRKT computation is 4 × 4 × 4, unless otherwise specified in the paper.

The first test aimed to demonstrate the significant advantage of the proposed IRKT over the classical Krawtchouk transform. As explained in Section 3, the IRKT is a lossless and reversible transform, ensuring that images reconstructed using the IRKT are indistinguishable from the original images. Table 4 presents the average MSE and PSNR values for medical images reconstructed using the IRKT. These results hold true regardless of the value assigned to the Krawtchouk polynomial parameter p within the range of p∈(0,1). Additionally, we compared the IRKT with the classical Krawtchouk transform when *p* = 0.5, as this value captures the maximum image information [13]. Notably, the calculations for these transforms were performed using the long format specified by the IEEE^®^ standard for floating-point arithmetic. Table 4 clearly shows that medical images reconstructed using the proposed IRKT are exactly identical to the original images. In contrast, the classical Krawtchouk transform lacks reversibility, preventing an exact reconstruction of the original images.

**Figure 4:**
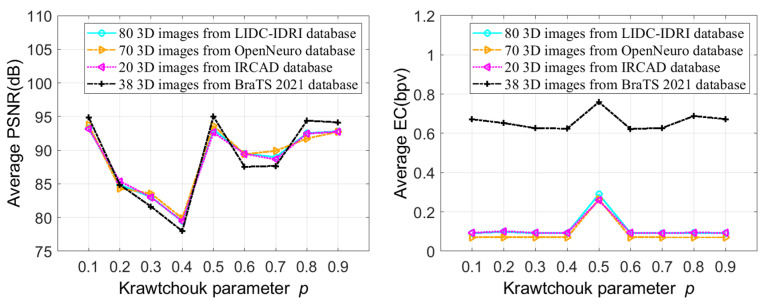
Average PSNR and EC obtained depending on the Krawtchouk parameter *p*.

In the second test, we investigated the impact of varying the Krawtchouk polynomial parameter *p* on the embedding capacity and quality of stego images. For each test image, the IRKT was applied, and then the additional data were inserted into the IRKT domain using the threshold *T* = 1. Subsequently, the stego image was generated by applying the inverse IRKT. In this test, the IRKT was implemented by increasing the Krawtchouk polynomial parameter *p* from 0.1 to 0.9 with an increment of 0.1. Figure 4 shows the average PSNRs and ECs of stego images for these different Krawtchouk polynomial parameters. It can be seen from Figure 4 that the parameters *p* = 0.1, 0.5, 0.8 and 0.9 are comparable and give high additional data imperceptibility (high PSNR) compared to the other *p* values. On the other hand, the Krawtchouk parameter *p* = 0.5 gives the highest EC. This is justified by the fact that *p* = 0.5 provided an IRKT histogram more concentrated on the interval [−*T*, *T*) with significantly high peaks in this interval. And as the capacity of our method is dependent on the number of IRKT coefficients in the interval [−*T*, *T*), we can embed more additional data with the parameter *p* = 0.5 and therefore a high embedding capacity. In the following tests, the Krawtchouk polynomial parameter *p* = 0.5 is used for calculating IRKT and inverse IRKT.

**Figure 5:**
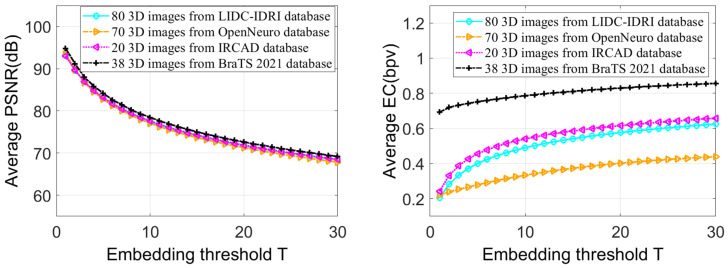
Average PSNR and EC obtained depending on the embedding threshold *T*.

In the third test, we analyzed the influence of threshold *T* variation on IRKT-based RDH performance. The IRKT was applied to each test image, then the additional data were embedded in the IRKT domain with threshold *T* values ranging from 1 to 30. Figure 5 presents the average PSNR and EC of the stego images under these different threshold values. Figure 5 illustrates that as the threshold value *T* increases, stego images can carry more additional data, because a higher *T* value reserves more space in the IRKT domain for embedding the additional data bits. However, raising the threshold *T* results in a reduction in the PSNR value for the stego images. This is because the IRKT coefficients undergo a *T*-position shift, leading to more significant modifications when using higher *T* values. The figure also demonstrates that there is a notable variation in PSNR and EC for lower threshold values, but this variation gradually diminishes as the thresholds increase. Eventually, at a given threshold, the variation in PSNR and EC becomes nearly negligible. For example, when increasing the threshold from *T* = 1 to *T* = 5 for LIDC-IDRI images, the average PSNR and EC values changed from (93.06 dB, 0.2061 bpv) to (83.01 dB, 0.4008 bpv), respectively, resulting in a variation of 10.05 dB and 0.1947 bpv, respectively. Conversely, when increasing the threshold from *T* = 25 to *T* = 30, the average PSNR and EC values changed from (69.90 dB, 0.6037 bpv) to (68.39 dB, 0.6250 bpv), respectively, resulting in a variation of 1.51 dB and 0.0213 bpv, respectively.

Table 5, Table 6, Table 7 and Table 8 show the average results (PSNR and EC) over thresholds ranging from *T* = 1 to *T* = 30, for various stego medical images. These tables clearly show that the IRKT-based 3D HDR algorithm offers a high data embedding capacity while preserving a high PSNR.

**Figure 6:**
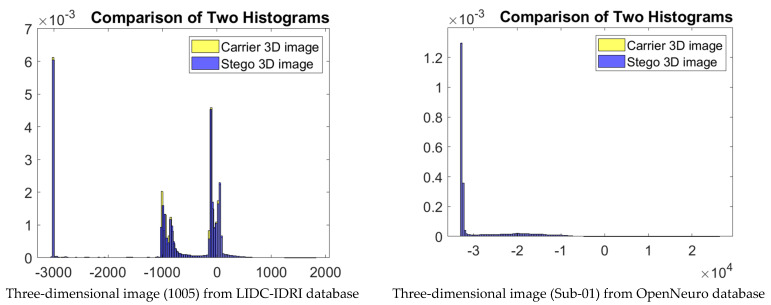

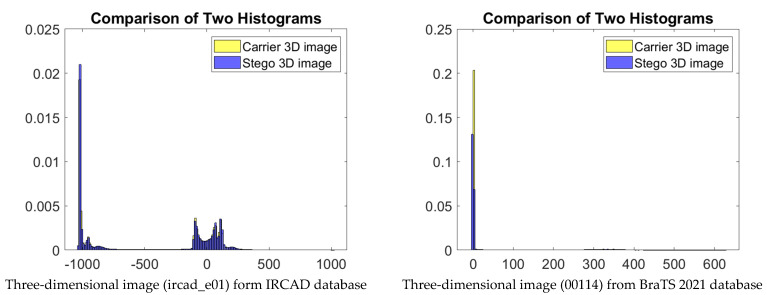
Histogram-based comparison of many different types of medical images and their stego versions.

In the fourth test, the robustness of the proposed IRKT-based RDH algorithm against statistical attacks was tested. Here, robustness against statistical attacks refers to the ability of the method to resist attempts at statistical analysis or detection of additional data that are hidden inside an image. Figure 6 shows a histogram-based comparison of many different types of medical images and their stego versions. We can clearly observe from this figure the great similarity between the two histograms, which is an indication of the robustness of our method against the statistical attacks.

In order to quantitatively measure the difference between the histogram distributions of the two images, we use the Bhattacharyya distance [59]. It quantifies the statistical distance or divergence between the distributions, indicating how much they overlap or differ from each other. The Bhattacharyya distance ranges from 0 to infinity. A smaller distance indicates a higher similarity between the histograms, while a larger distance indicates greater dissimilarity. Note that the Bhattacharyya distance is defined between two probability distributions. Therefore, it is important to normalize the histograms to ensure that they represent valid probability distributions before computing the distance. Table 9 presents the average Bhattacharyya distance between the histogram distributions of the original and stego images from four medical databases, using thresholds ranging from *T* = 1 to *T* = 30. The small Bhattacharyya distances shown in Table 9 clearly indicate the high similarity between the histograms of the stego images and the original images. This similarity implies that the statistical properties of the image remain largely unchanged after the additional data have been embedded, making it difficult for an observer or a system to detect the presence of hidden information through statistical analysis alone.

**Figure 7:**
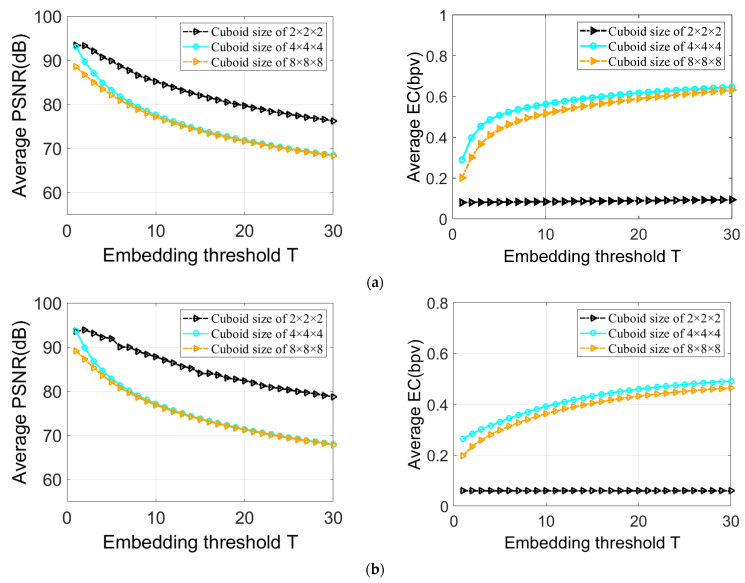

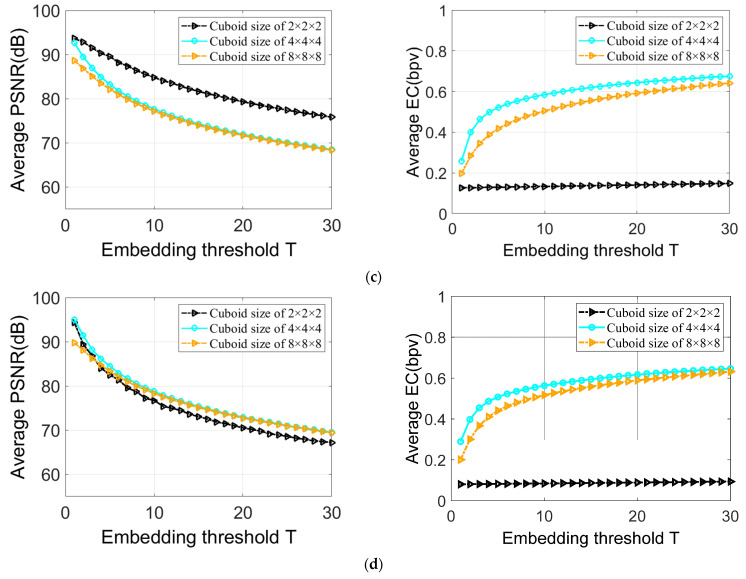
Average PSNR and EC using different cuboid sizes when calculating IRKT: (**a**) Results based on 80 3D images from LIDC-IDRI database; (**b**) results based on 70 3D images from OpenNeuro database; (**c**) results based on 20 3D images from IRCAD database; (**d**) results based on 38 3D images from BraTS 2021 database.

The fifth conducted test shows that the performance of the IRKT-based 3D RDH is improved by the cuboid size used when calculating IRKT. Figure 7 compares the PSNR and EC values of four medical databases, using cuboids of size 2 × 2 × 2, 4 × 4 × 4, and 8 × 8 × 8, with thresholds ranging from *T* = 1 to *T* = 30. From this figure, it is observed that the cuboid of size 4 × 4 × 4 offers better embedding capacity for all thresholds, followed by the cuboid of size 8 × 8 × 8 due to their more concentrated IRKT histograms in the interval [−*T*, *T*) and the presence of significantly higher peaks in this interval. On the other hand, the cuboid of size 2 × 2 × 2 shows a low embedding capacity for all thresholds due to its less-concentrated IRKT histogram in the interval [−*T*, *T*).

In terms of 3D stego image quality, the cuboid of size 4 × 4 × 4 offers a relatively high PSNR compared with the cuboid of size 8 × 8 × 8, especially for lower thresholds, while maintaining a comparable PSNR for higher thresholds. In contrast, the cuboid of size 2 × 2 × 2 has the highest PSNR due to its more limited embedding capacity. Overall, the cuboid of size 4 × 4 × 4 offers the maximum embedding capacity and a satisfactory PSNR for all thresholds.

The sixth test was performed to showcase the losslessness and reversibility of the IRKT-based 3D RDH algorithm. The results are presented in Table 10, which displays the average MSE and PSNR between the carrier 3D images and the restored images after the extraction of the additional data. Notably, the table demonstrates that the restored 3D images are exactly the same as the original carrier images. This significant advantage stems from the reversible nature of the proposed IRKT, ensuring the exact recovery of the original 3D carrier image upon extraction of the additional data. It is important to note that this achievement cannot be achieved using the classical Krawtchouk transform due to its nonreversible nature.

To show the superiority of the proposed IRKT-based RDH algorithm, we compare it with other algorithms from the perspective of embedding capacity and stego image quality. Table 11 presents a comparison between the proposed algorithm and other existing 3D algorithms based on histogram modification reported in [29,30,31]. It can be seen that the proposed algorithm is not only suitable for medical images, but also has a much better average embedding capacity than the existing 3D methods selected for this comparison.

Table 12 presents a comparison between the proposed algorithm and a similar lossless algorithm proposed by Huang et al. [22] for high-quality medical images. From this table, our method seems to have slightly better image quality based on the provided average PSNR values compared to Huang’s method. Moreover, our method achieves a higher average embedding capacity compared to Huang’s method, indicating that our method can potentially store more data within the same unit of space.

Table 13 provides a comprehensive comparison between the IRKT histogram-modification-based method and several other methods across different domains. The domains considered in the analysis include the spatial domain [32,56], the standard DCT domain [57], the standard DWT domain [58], the integer DCT domain [10], and the integer wavelet transform domains, such as integer CDF transform [8], integer 2/6 transform [9], integer Haar transform [9], and integer 9/7-F transform [9]. The comparison was conducted using four well-known 2D images: Lena, Mandrill, Airplane, and Boats. The average EC and PSNR values for each method are presented in the last columns. The results in Table 13 demonstrate that our proposed algorithm (2D IRKT, histogram modification, threshold-based embedding technique) achieves a very high embedding capacity and good stego image quality. The EC and PSNR values are higher than almost all other published algorithms selected for comparison, indicating a preference for our algorithm. Additionally, our algorithm’s average stego image quality is relatively lower than that of [32,56], integer Haar [9], and integer 2/6 [9], but it is relatively high compared to the other selected algorithms.

## 6. Conclusions

In this paper, we have successfully addressed the limitations of the conventional floating-point Krawtchouk transform by introducing the IRKT. By generating integer-valued coefficients in the Krawtchouk domain, the IRKT aligns effectively with the exact representation commonly utilized in lossless image applications. Expanding upon the IRKT framework, we have introduced a novel 3D RDH algorithm designed for the secure storage and transmission of extensive medical data in the IoMT sector. Our proposed algorithm employs histogram modification within the IRKT domain and a threshold-based embedding technique to embed additional/secret data. The histogram modification technique within the IRKT domain optimizes the utilization of the available embedding space, while the threshold-based embedding technique adjusts the embedding capacity and quality of the resulting 3D stego image based on a selected integer threshold.

Through comprehensive experimental assessments, we have demonstrated the effectiveness, imperceptibility, and robustness of our algorithm against statistical attacks. The algorithm showcases a substantial embedding capacity and upholds the quality of the host images. By integrating our proposed algorithm into the IoMT sector, we contribute enhanced security measures that guarantee the safe storage and transmission of extensive medical data.

Looking ahead, there are several exciting research avenues that warrant exploration. One particularly intriguing direction involves applying the IRKT in lossless compression, thereby further optimizing data storage and transmission efficiency. Furthermore, the incorporation of the IRKT into medical image encryption holds significant potential for reinforcing data privacy and security. Delving deeper into areas such as RDH within encrypted domains, stereo image RDH, and other relevant research domains could potentially yield valuable insights and drive notable advancements. Concurrently, we are dedicated to reducing the execution times of IRKT-based algorithms, particularly in scenarios where real-time or near-real-time processing is crucial. To achieve this, our strategy involves harnessing the capabilities of parallel computing techniques and leveraging the progress achieved in hardware acceleration methods [51,60], capitalizing on the advancements in these areas.

## Figures and Tables

**Table 1:** Row vectors for computing the SERMs of 4 × 4 ***K*** matrix.

$s_{0}^{T}$ = [	0.63299	−1.00113	1.00113	0	]
$s_{1}^{T}$ = [	0	−0.25951	0.25951	−0.61237	]
$s_{2}^{T}$ = [	0.12976	0	0.22474	0.43301	]
$s_{3}^{T}$ = [	0.68330	−0.81650	0	0.35355	]
$s_{4}^{T}$ = [	0.93265	−0.57735	−1.09638	0	]

**Table 2:** Row vectors for computing the SERMs of 8 × 8 ***K*** matrix.

$s_{0}^{T}$ = [	0.912366	−1.293027	−1.738221	−1.857055	1.923238	4.164748	4.787527	0	]
$s_{1}^{T}$ = [	0	−0.478498	−1.251264	−1.236242	1.270850	2.520126	2.305815	−0.522913	]
$s_{2}^{T}$ = [	0.035496	0	0.836863	0.453748	−1.166437	−1.687982	−1.561738	0.423608	]
$s_{3}^{T}$ = [	0.025099	−0.129757	0	0.320848	−0.824795	−0.785335	−1.811422	0.299536	]
$s_{4}^{T}$ = [	0.022913	0.527046	0.205163	0	−0.045825	−1.629780	−1.653595	0.273437	]
$s_{5}^{T}$ = [	−0.021909	0.150701	0.181791	0.449090	0	1.558368	1.581139	−0.261456	]
$s_{6}^{T}$ = [	0.750597	−0.709915	0.848661	0.782428	−0.522623	0	2.956796	−0.286411	]
$s_{7}^{T}$ = [	0.612860	0.142044	0.442929	0.079832	0.234718	−0.239146	0	−0.233854	]
$s_{8}^{T}$ = [	−1.775545	−2.571369	−0.004222	0.503776	−2.003696	−0.941328	6.166002	0	]

**Table 3:** Summary of 208 grayscale 3D medical images used as carrier images.

Database	Number of 3D Images	Body Part Examined	Modality	Format and Size	Class and Bit Depth
LIDC-IDRI [52]	80	Human lungs	CT	DICOM	Signed
				160 × 160 × 160	16 bit
OpenNeuro [53]	70	Head	MRI	NIfTI	Signed
				192 × 192 × 192	16 bit
IRCAD [54]	20	Liver	CT	NIfTI	Signed
				128 × 128 × 128	16 bit
BraTS 2021 [55]	38	Brain	MRI	DICOM	Unsigned
				96 × 96 × 96	16 bit

**Table 4:** Average error between reconstructed 3D images and original ones using IRKT and classical Krawtchouk.

3D Medical Images Database	Metric	Proposed IRKT for p∈(0,1)	Classical Krawtchouk Transform for *p* = 0.5
80 images from LIDC-IDRI [52]	PSNR (dB)	∞	341.5722
	MSE	0	2.9904 × 10^−25^
70 images from OpenNeuro [53]	PSNR (dB)	∞	312.5929
	MSE	0	2.3640 × 10^−22^
20 images from IRCAD [54]	PSNR (dB)	∞	343.7595
	MSE	0	1.8072 × 10^−25^
38 images from BraTS 2021 [55]	PSNR	∞	360.6442
	MSE	0	3.7028 × 10^−27^

**Table 5:** Average results for seven signed 16-bit 3D CT images from LIDC-IDRI database for all thresholds.

Image Name from the Database	Average EC (bits)	Average EC (bpv)	Average PSNR (dB)
1003	2,156,809	0.5266	75.7744
1005	2,125,935	0.5190	75.7660
1009	2,207,080	0.5388	75.8119
1045	2,191,740	0.5351	75.7590
1067	2,190,836	0.5349	75.7844
1201	2,037,064	0.4973	75.7113
1219	2,115,337	0.5164	75.7513

**Table 6:** Average results for seven signed 16-bit 3D IRMs from OpenNeuro database for all thresholds.

Image Name from the Database	Average EC (bits)	Average EC (bpv)	Average PSNR (dB)
sub-01	2,434,181	0.3439	75.3041
sub-02	2,271,334	0.3209	75.2472
sub-08	2,944,946	0.4161	75.4962
sub-09	2,649,708	0.3744	75.3851
sub-10	2,538,429	0.3586	75.3416
sub-11	2,641,653	0.3732	75.3831
sub-12	2,664,451	0.3764	75.3923

**Table 7:** Average results for seven signed 16-bit 3D CT images from IRCAD database for all thresholds.

Image Name from the Database	Average EC (bits)	Average EC (bpv)	Average PSNR (dB)
ircad_e01	1,127,532	0.5376	75.8147
ircad_e02	1,216,511	0.5801	75.9162
ircad_e03	1,084,896	0.5173	75.7673
ircad_e04	1,222,973	0.5832	75.8694
ircad_e05	1,149,071	0.5479	75.8471
ircad_e06	1,138,679	0.5430	75.8361
ircad_e07	1,217,345	0.5805	75.9177

**Table 8:** Average results for seven signed 16-bit 3D IRMs from BraTS 2021 database for all thresholds.

Image Name from the Database	Average EC (bits)	Average EC (bpv)	Average PSNR (dB)
00114	759,584	0.8585	76.9145
00119	707,022	0.7991	76.6859
00125	786,924	0.8894	77.0169
00153	784,908	0.8872	77.0111
00161	716,177	0.8095	76.7315
00174	704,289	0.7960	76.6777
00181	736,130	0.8320	76.8149

**Table 9:** The difference between the histogram distributions of carrier images and the stego images using Bhattacharyya distance.

IRKT-Based 3D RDH Method with	80 Images from LIDC-IDRI	70 Images from OpenNeuro	20 Images from IRCAD	38 Images from BraTS 2021
For *T* = 1	1.4416 × 10^−7^	7.8335 × 10^−7^	2.8873 × 10^−5^	2.2724 × 10^−6^
For all thresholds	3.49 × 10^−2^	1.2647 × 10^−4^	8.87 × 10^−2^	4.9249 × 10^−4^

**Table 10:** Average MSE and PSNR of the images restored after additional data extraction.

3D Medical Image Database	MSE	PSNR (dB)
80 3D images from LIDC-IDRI database	0	∞
70 3D images from OpenNeuro database	0	∞
20 3D images from IRCAD database	0	∞
38 3D images from BraTS 2021 database	0	∞

**Table 11:** Comparison between the proposed algorithm and histogram-modification-based 3D RDH algorithms [29,30,31].

Algorithm	Embedding Domain	Type of Carrier Data	Suitable for Medical Image	Average EC
[31]	Spatial	3D image models	No	0.2460 bits/vertex
[30]	Spatial	3D image models	No	0.1725 bits/vertex
[29]	Spatial	3D image models	No	0.2118 bits/vertex
Our	IRKT	3D grayscale medical image	Yes	0.4228 bits/voxel

**Table 12:** Comparison between the proposed algorithm and a similar lossless algorithm [22] for high-quality medical images.

Method	Embedding Domain	Unsign 16-bit Grayscale Medical Image		Sign 16-bit Grayscale Medical Image	
		Average EC	Average PSNR	Average EC	Average PSNR
[22]	Spatial	0.248 bits/pixel	74.66	0.124 bits/pixel	74.77
Proposed	IRKM	0.803 bits/voxel	76.70	0.422 bits/voxel	75.51

**Table 13:** Comparison between our proposed method and other RDH methods [8,9,10,32,56,57,58] for some commonly used images.

Method	Embedding Domain	Lena		Mandrill		Airplane		Boat		Average	
		EC (bits)	PSNR	EC (bits)	PSNR	EC (bits)	PSNR	EC (bits)	PSNR	EC (bits)	PSNR
[32]	Spatial	5460	48.20	5421	48.20	16,171	48.3	7301	48.20	8588.3	48.2250
[56]	Spatial	8835	48.2	5423	48.20	23,199	48.3	10,217	48.30	11,919	48.25
[8]	CDF transform	52,429	46.23	26,214	43.45	-----	-----	-----	-----	39,322	44.8400
[9]	Integer Haar	40,108	48.39	13,107	48.21	57,147	48.53	27,787	48.33	34,537	48.3650
[9]	Integer 2/6 transform	44,827	48.25	14,680	48.03	61,604	48.36	31,195	48.22	38,077	48.2150
[9]	Integer 9/7-F	50,070	45.25	17,564	45.46	67,633	45.35	35,914	45.26	42,795	45.3300
[10]	Integer DCT	64,345	41.787	23,904	41.505	93,951	42.039	50,848	41.697	58,262	41.7570
[57]	Standard DCT	36,857	30.34	35,389	26.46	36,805	29.98	36,700	29.75	36,438	29.1325
[58]	Standard DWT	39,872	30.08	-----	-----	39,453	27.84	39,138	31.41	39,488	29.7767
Proposed	IRKT	80,569	45.978	19,982	46.030	63,374	45.986	70,263	46.016	58,547	46.0031

## Data Availability

Data are available upon reasonable request.
